# Incidence, follow-up and outcomes of incidental abdominal aortic aneurysms in computed tomography

**DOI:** 10.1093/icvts/ivab319

**Published:** 2021-11-11

**Authors:** Fehim Can Sevil, Mehmet Tort, Çiğdem Özer Gökaslan, Hülya Sevil, Necip Becit

**Affiliations:** 1 Department of Cardiovascular Surgery, Afyonkarahisar Health Sciences University Hospital, Afyonkarahisar, Turkey; 2 Department of Radiology, Afyonkarahisar Health Sciences University Hospital, Afyonkarahisar, Turkey; 3 Department of Emergency, Afyonkarahisar Health Sciences University Hospital, Afyonkarahisar, Turkey

**Keywords:** Abdominal aortic aneurysm, Computed tomography, Diagnosis

## Abstract

**OBJECTIVES:**

The goal of our study was to determine the prevalence of abdominal aortic aneurysms (AAAs) that were incidentally diagnosed by computed tomography applied for different reasons and to discuss the risk factors that may cause AAA.

**METHODS:**

A total of 5396 abdominal computed tomography examinations were performed, and the 103 incidentally detected AAAs were included in the study. Patients with and without AAA were compared in terms of age, gender, thoracic and abdominal aortic diameters and comorbid diseases.

**RESULTS:**

The prevalence of the AAAs was 1.9%. Old age and male gender were significantly different between the groups (*P* < 0.001). The reason for applying computed tomography in 52 (50.5%) patients with AAA was associated with malignancy. In the evaluation of all patients in the study, the aortic diameter was determined to be larger in patients with malignancy than in patients without malignancy (18.07 ± 4.1 mm vs 17.7 ± 3.9 mm, respectively; *P* < 0.001). The thoracic aortic diameter was wider in patients with AAA compared to that in patients without AAA (37.2 ± 3.9 mm vs 33.9 ± 5.2 mm, respectively; *P* < 0.001). The presence of coronary artery disease, diabetes mellitus, hypertension and a history of smoking in patients with AAA was significantly different from that of patients without AAA (*P* < 0.001). There was no significant difference between the groups in terms of hyperlipidaemia and chronic obstructive pulmonary disease (*P* = 0.52 and *P* = 0.15, respectively).

**CONCLUSIONS:**

Screening of older men with diseases such as malignancy, hypertension, diabetes mellitus and coronary artery disease for AAA is important for the early diagnosis and treatment of this disease.

**Figure:**
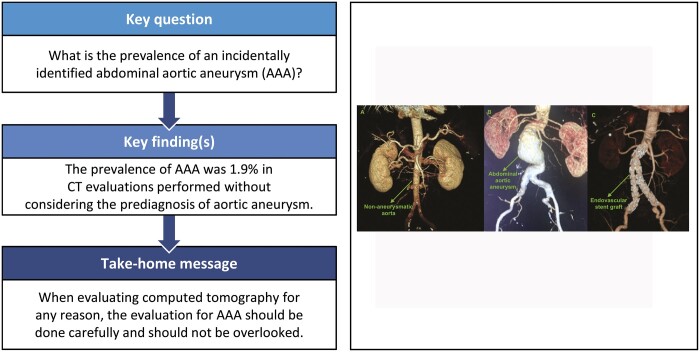

## INTRODUCTION

Abdominal aortic aneurysm (AAA) is difficult to diagnose because it is usually asymptomatic. It can be successfully treated with surgical intervention, but delay in diagnosis can lead to aortic rupture and life-threatening conditions [1]. Diagnosis of various incidental diseases with radiological imaging methods is common. AAA can be detected from computed tomography (CT) scans performed for any reason. Because the disease is progressive, such patients should be evaluated in particular. Incidentally detected, small AAA can be followed up serially, whereas the patients with large AAA can be treated with early interventions without development of any complications [2].

Our goal was to determine the prevalence of AAA in CT performed for different reasons in our hospital and to discuss the characteristics of the patients.

## MATERIALS AND METHODS

### Ethical statement

The study protocol adhered to the guidelines stipulated in the Declaration of Helsinki and was approved as an electronic medical record-based retrospective study by the ınstitutional review board of Afyonkarahisar Health Sciences University Hospital (05.05.2020/2011-KAEK-2); as such, the requirement for obtaining informed consent from the patients prior to study participation was waived.

A total of 5396 contrast and non-contrast abdominal CT examinations performed between January 2020 and June 2020 for any reason were obtained from the hospital information system and analysed retrospectively. A total of 103 incidentally detected AAAs were included in the study. Patients under the age of 18, those with a previous diagnosis of AAA, an intervention due to AAA, a previous diagnosis of vascular disease and abdominal pain in whom a pulsatile mass is detected in the abdomen on physical examination and those in whom the entire abdominal aorta could not be evaluated were excluded from the study.

In all abdominal CT examinations evaluated, the section with the largest aortic diameter was enlarged 3 times, and axial and sagittal anteroposterior aortic measurements were made using an electronic calliper tool. Measurements were performed using the line passing through the centre of the aorta from the aortic outer adventitia to the opposite outer adventitia. AAA was defined as having a diameter of 30 mm or more in the widest part of the aorta.

The demographic characteristics, reasons for CT application and comorbid diseases of the patients were analysed by scanning their files through the hospital system. Comorbidities such as hyperlipidaemia, diabetes mellitus, hypertension and chronic obstructive pulmonary disease were evaluated by means of the information obtained from the anamnesis of the patients.

Patients with an aortic diameter between 30 and 55 mm were followed up and were informed about their diseases. Endovascular aortic repair (EVAR) was applied to patients with an aortic diameter greater than 55 mm. Patients who underwent EVAR were also followed up with CT.

Patients with and without AAA were compared in terms of age, gender, thoracic and abdominal aortic diameters and comorbid diseases.

### Statistical analyses

Categorical variables were summarized as frequencies and percentages, whereas continuous variables were summarized as the median with interquartile ranges or the mean and the standard deviation. The normality of the parameters was tested with the Kolmogorov–Smirnov test. The Fisher exact test or the χ^2^ test was used to compare patient characteristics and risk factors between AAA (+) and AAA (−) groups in the case of categorical variables, whereas the Mann–Whitney *U*-test or the Student’s *t*-test was applied for continuous variables as appropriate. All statistical analyses were performed using the R statistical software version 3.2.2 (R Foundation for Statistical Computing, Vienna, Austria). A *P*-value of <0.05 was considered to indicate statistical significance.

## RESULTS

During CT application, 5396 patients with a mean age of 53.7 ± 17 years (range 18–90 years) were screened; 2643 (49%) of the patient population were men. The most common reason for CT scans was abdominal pain, seen in 1985 (31.2%) patients. The second most common reason for CT scans was malignancy screening or follow-up in 1681 (31.2%) of the patients. The mean axial aortic diameter of all patients was 17.6 ± 4 mm (range, 10.21–86.95 mm). Diabetes mellitus was present in 1122 (20.8%) of the screened patients, and it was the most common comorbid disease. Hyperlipidaemia was the second most common, detected in 1019 patients (18.9%). Of these patients, 852 (15.8%) had hypertension and 259 (4.8%) had chronic obstructive pulmonary disease. A total of 339 (6.3%) patients used statins during the screening. Although 4074 (75.5%) patients did not mention smoking in their history, 869 (16.1%) were ex-smokers and 453 (8.4%) were active smokers. Of the patients screened, 1753 (32.5%) had a diagnosed malignancy. Patient characteristics are shown in Table 1. A total of 1681 patients were screened for malignancy; no malignancy was detected in some of these patients. Also, some patients who were not initially examined for malignancy were diagnosed with malignancy after the CT scan. Of the patients with malignancy who underwent CT scans for reasons such as abdominal pain, trauma and traffic accidents, 1681 of them underwent CT for malignancy screening, whereas 1753 of the patients had comorbidities with malignancy.

**Table 1:** Baseline characteristics of the 5396 patients in the study group

Characteristic	Mean ± SD or No. (%)
Age, years (mean ± SD)	53.7 ± 17
Male	2643 (49)
Mean abdominal aortic diameter (mm)	17.6 ± 4
Hyperlipidaemia	1019 (18.9)
Diabetes mellitus	1122 (20.8)
Hypertension	852 (15.8)
Chronic obstructive pulmonary disease	259 (4.8)
Use of statin	339 (6.3)
Smoking	
Never	4074 (75.5)
Ex	869 (16.1)
Current	453 (8.4)
Malignancy	1753 (32.5)

SD: standard deviation.

**Figure 1:**
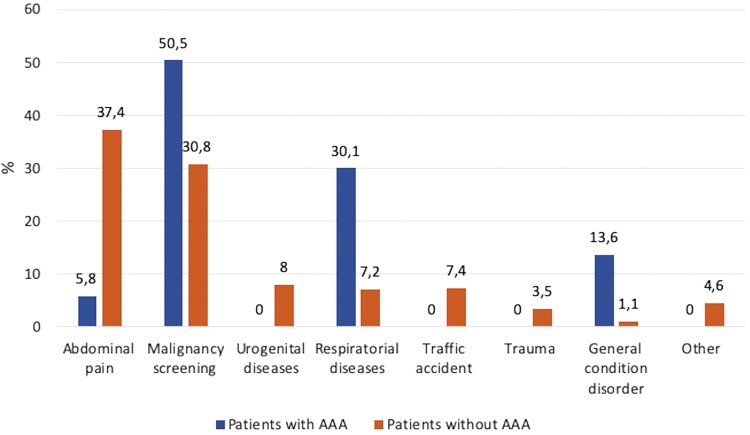
The reason for examining all patients with computed tomography. Values are given with ‘%’. AAA: abdominal aortic aneurysm.

In 103 (1.9%) of 5396 patients who were screened, the abdominal aortic diameter was found to be greater than 30 mm (range 10.21–86.95 mm). The mean age of patients with AAA was 71.8 ± 8.7 years, and 87 (84.5%) of these patients were men. When compared with patients without AAA, age and male gender were found to be significantly different between both groups (*P* < 0.001). AAA was diagnosed mostly in patients who were examined for malignancy (*n* = 52, 50.5%). These patients were known to have a previous malignancy, and the reason for CT was screening for malignancy. AAA was diagnosed in 6 patients (5.8%) who were examined for abdominal pain. Thirty-one patients (30.1%) with AAA had the CT scans for respiratory system evaluation; 14 (13.6%) patients diagnosed with AAA underwent CT because of a general disorder (Fig. 1).

The thoracic aortic diameters of patients with AAA were found to be significantly higher than those of patients without AAA (37.2 ± 3. 9 mm vs 33.9 ± 5.2 mm; *P* < 0.001, respectively). Whether there was an aortic calcification was detected by CT scan. The degree of stenosis on the lumen was determined to be the ratio of the distance through the middle of the lumen and the opposing lumen walls to the distance through the middle of the aorta in the same aortic segment and from the aortic wall to the opposite wall. Concurrently, aortic calcification and stenosis over 30% in the aortic lumen were observed to be more common in patients with AAA than in patients without AAA during the examinations performed to view the aortic lumen (*P* < 0.001).

**Figure 2:**
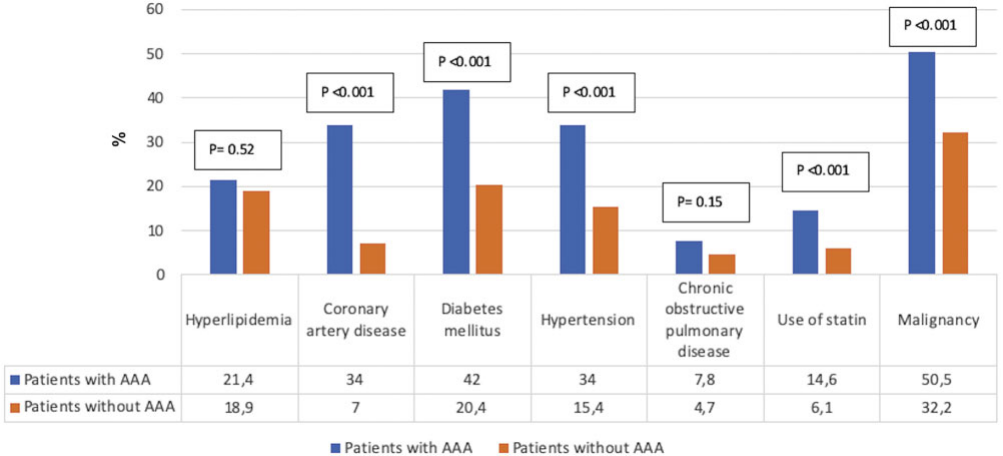
The comparison of the characteristics of the patients according to the comorbities. Values are given with ‘%’. AAA: abdominal aortic aneurysm.

Whereas coronary artery disease (*n* = 35, 34%), diabetes mellitus (*n* = 43, 42%), hypertension (*n* = 35, 34%) and smoking were more common in patients with AAA (*P* < 0.001), hyperlipidaemia (*n* = 22, 21.4%; *P* = 0.52) and chronic obstructive pulmonary disease (*n* = 8, 7.8%; *P* = 0.15) were not different between the 2 groups. The use of statins by patients with AAA was significantly lower than in patients without AAA (*n* = 15, 14.6% vs *n* = 322, 6.1%; *P* < 0.001, respectively) (Fig. 2). The mean creatinine value of the patients with AAA was 1.4 ± 0.8 mg/dl, whereas the same value was 0.9 ± 0.6 mg/dl (*P* < 0.001) in patients without AAA. In our clinic, CT images of all patients with AAA were evaluated by the radiology clinic in terms of malignancy. We found that 52 (50.5%) of the patients with AAA had malignancy and 1701 (32.2%) of the patients without AAA had malignancy; the difference between the 2 groups was significant (*P* < 0.001; Fig. 2).

At the follow-up examination, 24 (23.3%) of the patients with AAA had died; 174 (3.3%) of the patients without AAA had died; the difference in the number of deaths between the 2 groups was significant. The patients with AAA did not die of AAA; rather they died of general condition disorders, malignancy and advanced respiratory diseases. A comparison of the characteristics of the patients according to the presence of AAA is given in Table 2.

**Table 2:** Comparison of the characteristics of the patients according to the presence of an abdominal aortic aneurysm

Characteristic	Patients with AAA (*n* = 103)	Patients without AAA (*n* = 5293)	*P-value*
Age, years (mean ± SD)	71.8 ± 8.7	53.38 ± 17	<0.001
Male	87 (84.5%)	2556 (48.3%)	<0.001
Mean thoracic aortic diameter (mm)	37.2 ± 3.9	33.9 ± 5.2	<0.001
Aortic calcification	103 (100%)	1688 (31.9%)	<0.001
Aortic occlusion	6 (5.8%)	95 (1.8%)	<0.001
Smoking			
Never	43 (42.2%)	4033 (76.2%)	<0.001
Ex	54 (51.5%)	815 (15.4%)	
Current	6 (5.8%)	445 (8.4%)	
Creatinine (mg/dl)	1.4 ± 0.8	0.9 ± 0.6	<0.001
Deaths	24 (23.3%)	174 (3.3%)	<0.001

AAA: abdominal aortic aneurysm; SD: standard deviation.

The mean age of 1753 patients with malignancy, with and without AAA, was 60 ± 12.5 years; 948 (54%) of these patients were men (*P* < 0.001). In patients with malignancy, the abdominal aortic diameter was found to be significantly higher than that in patients without malignancy (18.07 ± 4.1 mm vs 17.7 ± 3.9 mm; *P* < 0.001, respectively). It was determined that the thoracic aorta, like the abdominal aorta, was larger in patients with malignancy (34.3 ± 4.7 mm vs 33.7 ± 5.5 mm, respectively; *P* < 0.001). A comparison of patients with and without malignancy is given in Table 3.

**Table 3:** Comparison of some aspects of patients with malignancy and patients without malignancy

Characteristic	Patients with malignancy (*n* = 1753)	Patients without malignancy (*n* = 3643)	*P-value*
Age, years (mean ± SD)	60 ± 12.5	50.7 ± 18	<0.001
Male	948 (54%)	1686 (46%)	<0.001
Mean abdominal aortic diameter (mm)	18.07 ± 4.1	17.7 ± 3.9	<0.001
Mean thoracic aortic diameter (mm)	34.3 ± 4.7	33.7 ± 5.5	<0.001
Aortic calcification	761 (43.4%)	1014 (27.8%)	<0.001
Hyperlipidaemia	400 (22.8%)	616 (16.9%)	<0.001
Coronary artery disease	117 (6.6%)	289 (7.9%)	0.31
Diabetes mellitus	316 (18%)	809 (22.2%)	0.01
Hypertension	166 (9.4%)	690 (18.9%)	<0.001
Chronic obstructive pulmonary disease	102 (5.8%)	156 (4.2%)	0.002
Smoking			
Never	904 (51.5%)	3194 (87.6%)	<0.001
Ex	634 (36.1%)	211 (5.7%)	
Current	143 (8.1%)	308 (8.4%)	
Creatinine (mg/dl)	0.9 ± 0.5	0.9 ± 0.7	0.31

SD: standard deviation.

**Figure 3:**
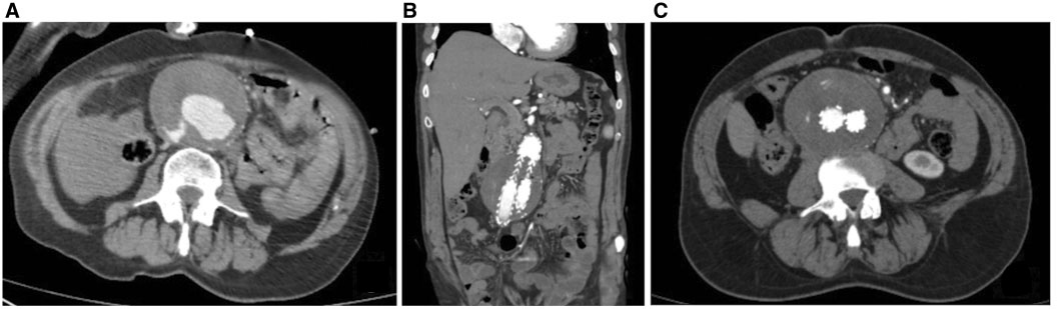
The computed tomography image of an abdominal aortic aneurysm. (**A**) A computed tomography image of a ruptured abdominal aortic aneurysm. (**B**) An image of endovascular repair of the aortic aneurysm repair. (**C**) The endovascular graft could seen in the aortic lumen in the same patient in the follow-up period; no adverse event was detected.

AAA was detected in 103 (1.9%) of the patients screened; these patients are grouped according to aortic diameters (Table 4). Patients were called for follow-up based on their aortic diameters and the rate of increase of the diameters. The mortality and morbidity of the patients were evaluated by scans of patients’ electronic files. Among the patients whose aortic diameter was between 30 and 39 mm, 82 (79.6%) had AAA; these patients were followed up annually. The aortic diameter was 40–44 mm in 14 (13.6%) patients with AAA; these patients were reviewed every 6 months. The aortic diameter was 45 to 54 mm in 3 (2.9%) patients with AAA; these patients were re-evaluated 1 month after the diagnosis and then every 6 months as long as the aneurysm was not enlarged. The aortic diameter was above 55 mm in 4 (3.9%) patients with AAA; aortic rupture was detected in 1 of these patients. EVAR was applied to all patients with an aortic diameter above 55 mm, and the aorta was evaluated with a follow-up CT at 1, 3 and 6 months after the procedure. We observed that the aneurysm sac was completely thrombosed, and there were no complications such as endoleaks (Fig. 3). No deaths associated with AAA were observed during the follow-up and treatment of the patients.

**Table 4:** Abdominal aortic aneurysm reporting for routine abdominal computed tomography scans according to aneurysm size

Abdominal aortic size, mm	*N* (%)
30–39	82 (79.6%)
40–44	14 (13.6%)
45–54	3 (2.9%)
≥55	4 (3.9%)

## DISCUSSION

In our study, the prevalence of AAA was 1.9% based on the CT evaluation performed without considering the pre-diagnosis of aortic aneurysm. The majority of patients with incidentally detected AAAs were patients with malignancy. It can be argued that we played an effective role in the survival of these patients because we performed EVAR on 4 patients with an aortic diameter above 55 mm. We are of the opinion that, thanks to the close follow-up of other patients with AAA, these patients benefitted greatly. Therefore, regardless of the purpose of imaging, patients should be evaluated for AAA, the results of which can be catastrophic and increase mortality and morbidity considerably.

The prevalence of AAA in patients over 65 years of age has been reported to be 1.3% [3]. Consistent with results of previous studies, the average age of patients with AAA over 65 years of age in our study revealed the need for a more careful evaluation in this patient group. It has been reported that 73% of patients diagnosed with incidental AAA are men [2–4]. Our study shows that 84.5% of the patients with AAA are men, which is consistent with the results of previous studies.

Although abdominal and back pains are common among AAA symptoms, AAA is primarily asymptomatic and the diagnosis of AAA is made incidentally [5]. Although only 5.8% of patients with AAA had abdominal pain, 50.5% of patients with AAA had malignancy. It has been stated in the studies that antimetabolite therapies can be effective in the development and progression of an aneurysm [6]. We think that a comprehensive study should be conducted on aortic pathologies in patients with malignancy.

AAA is usually accompanied by other vascular diseases. Thoracic aortic aneurysms may accompany AAA [7]. As shown in our study, thoracic aortic diameters in patients with AAA were found to be significantly higher than those in other patients (*P* < 0.001). Concurrently, the majority of patients with aortic calcification and stenosis of more than 30% in the aorta were those who were diagnosed with AAA (*P* < 0.001). Considering the data we have obtained, we think that detailed examinations of all vascular structures including the thoracic aorta of patients with AAA will be beneficial for these patients.

It was stated in previous studies that diabetes mellitus and hyperlipidaemia, which predispose to atherosclerosis, are not evident in AAA. Consistent with the data in the literature, other researchers found that coronary artery disease and hypertension among the vascular risk factors were more common in patients with AAA compared to patients without AAA (*P* < 0.001) [4, 8, 9]. Unlike previous studies, whereas diabetes mellitus was significantly more common in patients with AAA (*P* < 0.001), the effect of hyperlipidaemia on AAA was not shown in accordance with these studies (*P* = 0.52); however, the use of statins was found at a lower rate in patients with AAA (*P* = 0.001) [10]. In the light of the literature data and our results, we are of the opinion that the use of statins may be beneficial in the follow-up period and after treatment of AAA. Smoking is among the main risk factors for development of AAA [4, 11–13]. In our study, 57.3% of the patients with AAA were active smokers or had a history of smoking.

As stated in the studies conducted, AAA can be detected in small diameters in patients with malignancy. Although the tests performed for malignancy yielded this result, the growth rates of the aneurysm and the possibility of rupture are almost the same [6]. In our study, malignancy was common in older male patients (*P* < 0.001). The frequent occurrence of AAA in older male patients inevitably causes these 2 conditions to be common. In addition, some studies have established a direct relationship between malignancy and AAA [14]. In our study, abdominal aorta and thoracic aorta diameters were found to be significantly higher in patients with malignancy (*P* < 0.001). In previous studies, it was shown that the increase in aortic diameter was caused by biological signal pathways such as transforming growth factor-β and mitogen-activated protein kinase [15–17]. We are of the opinion that the reason for the larger diameter of the abdominal aorta and thoracic aorta in malignant patients is due to such biological signal pathways.

Changes in modifiable risk factors including smoking cessation and blood pressure control are recommended for patients with an AAA diameter <50 mm. Primary indications for intervention in patients with AAA include development of symptoms, rupture, rapid aneurysm growth (>5 mm in 6 months) or the presence of a fusiform aneurysm with a maximum diameter of 55 mm or greater [5]. The mortality rate associated with rupture is high and varies between 60% and 80%. Therefore, early diagnosis and treatment is important before a rupture occurs [11, 18]. Thanks to an increased number of screening methods, AAA-related mortality and morbidity have decreased significantly, and the chance of intervention before development of rupture in these patients has increased [19]. Intervention for AAA includes conventional open surgical repair and endovascular aortic stent graft repair. In our study, patients with an aortic diameter <55 mm were given medical treatment and followed up with intermittent imaging. EVAR was successfully performed in patients with AAA with an aortic diameter greater than 55 mm, and open surgical repair was not required. Post-procedure follow-up of these patients was done with CT; no complications such as leakage or aneurysm enlargement were observed. Our study has found no relation between the cause of mortality and the aortic aneurysm in patients with AAA who have died.

## CONCLUSION

An aortic aneurysm is a life-threatening condition; it is mostly asymptomatic and incidentally diagnosed. The survival rate can be increased with early diagnosis and early intervention. In particular, careful evaluation of older male patients and patients with a history of malignancy for AAAs via imaging methods performed for other reasons will allow early intervention in such patients.

### Limitation

Our study has limitations due to the multifactorial nature of the CT application and the imbalance between the groups. We think that the use of analytic methods such as multivariable analysis and score matching among these groups will yield more valuable results. There is a need for more comprehensive and subgroup studies because some of these patients were screened for malignancy and some of these patients did not have malignancy as shown by the screening, as well as patients who had malignancies but who were screened for other reasons such as a traffic accident or abdominal pain.

**Conflict of interest:** none declared. 

## Data Availability Statement

Data cannot be shared for ethical reasons. The data underlying this article cannot be shared publicly due to the retrospective nature of the study and because the consent of the patients was not obtained for sharing. The data are contained in the closed electronic system of a public hospital. If data are requested, data can be provided by hiding patient information. However, the ethics committee is very determined to protect personal data. The data will be shared on reasonable request to the corresponding author.

## Author contributions

**Fehim Can Sevil:** Conceptualization; Data curation; Investigation; Methodology; Project administration; Writing—original draft. **Mehmet Tort:** Project administration; Resources; Software; Supervision; Visualization. **Çiğdem Özer Gökaslan:** Formal analysis; Investigation; Methodology; Project administration; Visualization. **Hülya Sevil:** Data curation; Investigation; Project administration; Validation; Visualization; Writing—original draft. **Necip Becit:** Funding acquisition; Methodology; Validation; Writing—review & editing.

## Reviewer information

Interactive CardioVascular and Thoracic Surgery thanks Yasunori Iida, Hirofumi Hioki and the other anonymous reviewers for their contribution to the peer review process of this article.

## ABBREVIATIONS

AAA: Abdominal aortic aneurysm
CT: Computed tomography
EVAR: Endovascular aortic repair
